# Agreement with evidence for tissue Plasminogen Activator use among emergency physicians: a cross-sectional survey

**DOI:** 10.1186/s13104-015-1242-5

**Published:** 2015-06-26

**Authors:** Alice M Grady, Jamie Bryant, Mariko L Carey, Christine L Paul, Rob W Sanson-Fisher, Christopher R Levi

**Affiliations:** Priority Research Centre for Health Behaviour, Hunter Medical Research Institute (HMRI), University of Newcastle, Callaghan, NSW 2308 Australia; Department of Neurology, John Hunter Hospital, New Lambton, NSW 2305 Australia; Priority Research Centre for Translational Neuroscience and Mental Health, University of Newcastle, Callaghan, NSW 2308 Australia

**Keywords:** Emergency medicine, Stroke, Stroke care, Thrombolysis, Treatment

## Abstract

**Background:**

Emergency department staff play a crucial role in the triage of stroke patients and therefore the capacity to deliver time-dependent treatments such as tissue Plasminogen Activator. This study aimed to identify among emergency physicians, (1) rates of agreement with evidence supporting tissue Plasminogen Activator use in acute stroke care; and (2) individual and hospital factors associated with high agreement with evidence supporting tissue Plasminogen Activator use.

**Methods:**

Australian fellows and trainees of the Australasian College for Emergency Medicine were invited to complete an online cross-sectional survey assessing perceptions of tissue Plasminogen Activator use in acute stroke. Demographic and hospital characteristics were also collected.

**Results:**

429 Australasian College for Emergency Medicine members responded (13% response rate). Almost half (47.2%) did not agree with any statements regarding the benefits of tissue Plasminogen Activator use for acute stroke. Perceived routine administration of tissue Plasminogen Activator by the head of respondents’ emergency department was significantly associated with high agreement with the evidence supporting tissue Plasminogen Activator use in acute stroke.

**Conclusions:**

Agreement with evidence supporting tissue Plasminogen Activator use in acute stroke is not high among responding Australian emergency physicians. In order for tissue Plasminogen Activator treatment to become widely accepted and adopted in emergency settings, beliefs and attitudes towards treatment need to be in accordance with clinical practice guidelines.

**Electronic supplementary material:**

The online version of this article (doi:10.1186/s13104-015-1242-5) contains supplementary material, which is available to authorized users.

## Background

### Stroke and tissue Plasminogen Activator (tPA)

Globally, stroke is the third most common cause of disability-adjusted life years (DALYs) [1]. Thrombolytic therapy using intravenous tPA is both an effective [2] and cost-effective [3] treatment in eligible acute ischemic stroke patients, significantly improving the chance of a good recovery when used in accordance with strict protocols [4]. However, tPA carries risk of haemorrhage [4] and can only be administered within 4.5 h of stroke onset [5], which provides challenges to its widespread use. Currently 53% of Australian hospitals report offering [6], and 7% of patients receive, tPA treatment [7]. Rates of use are lower in other countries; 5% of stroke patients receive tPA in the United Kingdom (UK) [8] and 2.4% of ischemic stroke patients receive tPA in the United States (US) [9].

### Physicians’ attitudes towards tPA

Emergency department (ED) staff play a crucial role in the triage and in-hospital care of stroke patients. Timely assessment and referral in line with clinical guidelines is particularly important for stroke patients eligible for tPA treatment. Emergency physicians may be responsible for the treatment of stroke patients in hospitals without dedicated specialists.

Risks associated with tPA in acute stroke may contribute to physician uncertainty in administering treatment [4]. A 2005 survey of US emergency physicians indicated 40% were unlikely to administer tPA for acute stroke in an ideal setting [10]. Attitudes may be shifting with a 2010 survey showing that 17% of emergency physicians were uncertain or unlikely to administer tPA in an ideal setting [11]. However, the latter survey was limited to emergency physicians from community hospitals participating in a cluster randomised controlled trial [11]. A 2004 New Zealand (NZ) survey indicated 73% of physicians rarely or never administer tPA treatment [12]. This sample included a range of health-care providers, limiting the generalisability of findings to the emergency context.

A discrepancy between the optimum hospital environment outlined in clinical guidelines and the actual settings in which tPA treatment is administered may also contribute to implementation challenges [6, 13]. Furthermore, in 2013, Australia’s National Stroke Foundation (NSF) reported 24% of hospitals without a stroke care unit (SCU) provide thrombolysis [6].

Emergency physicians’ perceptions of the evidence in support of tPA use in stroke have not been examined recently. Perceptions may have altered as a consequence of public media campaigns [F.A.S.T (Face, Arm, Speech, Time) campaigns launched by NSF, American Heart Association (AHA) and American Stroke Association (ASA), and the UK’s Stroke Association], publicised debate regarding potential benefits and risks of tPA treatment [14], and release of clinical guidelines with an increased time-frame for treatment [5, 15]. As such, an examination of whether attitudes of emergency health-care providers are supportive of tPA use in acute stroke, or whether they might be one of the factors limiting tPA rates is required. The study successfully identified, among a sample of emergency physicians, (1) rates of agreement with evidence supporting tPA use in acute stroke care; and (2) individual and hospital factors associated with high agreement with evidence supporting tPA use.

## Methods

### Setting

An online cross-sectional survey of emergency physicians in Australia was conducted July–August 2012. The University of Newcastle’s Human Research Ethics Committee and the Australasian College for Emergency Medicine (ACEM) Scientific Committee approved the study.

### Participants

Australian trainees and fellows registered with ACEM were invited to participate. All emergency physician trainees and fellows within Australia are registered with ACEM, providing access to a representative sample of this specialty group. Fellows of ACEM have completed a minimum of 7 years post-graduate medical training, and participate in ongoing professional training to maintain this title.

### Procedure

Potential participants were sent an email from ACEM containing an information statement and link to the survey. A reminder email was sent two weeks following initial correspondence. Completion of the survey was taken as implied consent.

### Measures

Responders completed an online survey administered via Survey Monkey (see Additional file 1). Measures were developed based on recommended hospital facilities and evidence supporting tPA use according to NSF’s Clinical Guidelines for Stroke Management [5]. Items were reviewed by two stroke specialists and two emergency physicians at a tertiary hospital to ensure they reflected findings from published literature. The survey was then pilot tested with five health behaviour researchers who reviewed the items to ensure comprehension. Items included:

#### Physician characteristics

Age; gender; role within the hospital; number of years worked in emergency care; and role in stroke care. De-identified data on all fellows and trainees (gender and location) was obtained from ACEM to assess response bias.

#### Hospital characteristics

State; whether arrangements for pre-hospital notification from ambulance are in place; number of ischemic stroke patients presenting to ED per fortnight; an estimate of the proportion of stroke patients referred to an SCU or neurology department; presence of an SCU; presence of an intensive care unit (ICU); whether advanced imaging facilities are available; whether the hospital provides tPA treatment; an estimate of the proportion of ischemic stroke patients receiving tPA treatment; an estimate of the proportion of emergency physicians giving tPA treatment; and whether the head of ED routinely provides tPA treatment.

#### Perception of tPA use in acute stroke

Participants were presented with six items reflecting published literature limited to the benefits of tPA use in acute stroke, and two items about their concerns using tPA treatment. Participants rated how much they agreed or disagreed with each item on a five point likert scale (strongly disagree [1] to strongly agree [5]). Participants were also asked ‘What would influence your views on the use of tPA in acute stroke?’ Response options included ‘Guidance from a professional colleague’, ‘Guidance from an expert in the use of tPA for acute stroke’, ‘Additional clinical trials of tPA’, ‘Research conducted by ED staff’, and ‘Other’.

### Data analysis

Descriptive statistics were calculated to describe the demographic and workplace characteristics of responders, and the survey responses. Characteristics of responders and grouped de-identified data on non-responders were compared using χ^2^ tests.

Each respondent received an accumulated score of 0–6 based on their level of agreement with items. For each item the responses ‘strongly agree’ or ‘agree’ were given a score of 1 and all remaining responses a score of 0. “High agreement” was defined as an accumulated score of ≥4; “Low agreement” was a score of 1–3; and “No agreement” was a score of 0. The association between all physician and hospital characteristics on the level of agreement (“High agreement” vs “Low/No agreement”) with evidence for tPA use in acute stroke was evaluated separately using logistic regression. All variables identified as significant at a p-value of 0.2 or less were included in a stepwise logistic regression analysis. For the final multiple logistic regression, variables that met a significance level of p < 0.05 were included in the model. Missing data were excluded.

## Results

The study had 55 and 52% power at the 5% significance level to detect a 0.55/0.45 difference in proportions of respondents who agree/disagree with each item.

### Response rate and physician characteristics

Of the 3,280 ACEM members invited to participate, 429 responded (response rate = 13%). Results of chi-square analyses indicated males [Χ^2^ (1, n = 3,278) = 6.54, p = 0.01], and respondents working in Victoria [Χ^2^ (7, n = 3,280) = 33.10, p < 0.01] were more likely to participate. Table 1 shows characteristics of responders and non-responders. Of those responsible for deciding which patients receive tPA, the median proportion of eligible patients perceived to be treated with tPA was 15%.

**Table 1:** Demographics and characteristics of responders (n = 370) and non-responders (n = 2910)

	Responders	Non-responders
Characteristic	Mean (SD)	Mean (SD)
Age	41.1 (8.2)	NA

	n (%)	n (%)
Male	256 (69.2)	1814 (62.4)
Years worked in emergency care		NA
≤5 years	59 (15.9)	
5–10 years	92 (24.9)	
11–15 years	90 (24.3)	
≥16 years	129 (34.9)	
Role within the hospital*		NA
Emergency physician	229 (63.8)	
Emergency physician trainee	122 (34.0)	
Other	8 (2.2)	
Location*		
New South Wales (NSW)	95 (26.5)	809 (27.7)
Victoria (VIC)	108 (30.1)	715 (24.5)
Queensland (QLD)	72 (20.1)	715 (24.5)
South Australia (SA)	20 (5.6)	212 (7.3)
Western Australia (WA)	27 (7.5)	331 (11.3)
Northern Territory (NT)	10 (2.8)	34 (1.2)
Australian Capital Territory (ACT)	8 (2.2)	44 (1.5)
Tasmania (TAS)	19 (5.3)	61 (2.1)
Responsible for determining care provided to stroke patients*	293 (81.6)	NA
Decide which patients receive tPA^†^	116 (41.7)	NA

Number of observations varies across characteristics due to missing data, multi-response items, and item applicability.

*NA* data not available.

* n = 359.

^†^n = 278.

### Hospital characteristics

Table 2 outlines participants’ self-reported hospital characteristics. The average number of ischaemic stroke patients seen by the ED every fortnight was 14.1 (SD 12.9). The median proportion of patients referred to a neurology department or SCU from emergency was perceived to be 85%. Of the hospitals that provide tPA treatment to eligible stroke patients, the median proportion of patients perceived to be treated with tPA by emergency physicians was 10%.

**Table 2:** Hospital characteristics of responders (n = 359)

Characteristic	N (%)
Arrangements are in place to receive pre-hospital notification of stroke patients from the ambulance service	238 (66.3)
The hospital has a dedicated stroke care unit*	266 (74.3)
The hospital has an intensive care unit*	338 (94.4)
The hospital has advanced imaging facilities (perfusion CT and MRI)*	300 (83.8)
The hospital provides tPA treatment to eligible ischemic stroke patients*	278 (77.7)
The proportion of the emergency physicians at the hospital who routinely administer tPA treatment for eligible ischemic stroke patients^†^	
None	184 (66.2)
Less than half	33 (11.9)
About half	8 (2.9)
Most	36 (12.9)
All	17 (6.1)
The head of the ED routinely administers tPA treatment for eligible ischemic stroke patients^†^	
Yes	32 (11.5)
No	173 (62.2)
I do not know	73 (26.3)

Number of observations varies across characteristics due to missing data and question applicability.

* n = 358.

^†^ n = 278.

### Physician agreement with evidence for tPA

**Figure 1:**
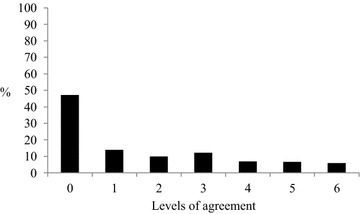
Emergency physicians’ level of agreement with evidence for tPA use (n = 415).

Physician agreement with statements for tPA use is provided in Table 3. There were no significant differences in rates of agreement between those who reported their hospital did or did not provide tPA. 19.5% of respondents had a high level of agreement with these statements, 36.2% had a low level, and 47.2% agreed with none of the statements (Figure 1).

**Table 3:** Physician agreement with the evidence for tPA use in acute stroke care (n = 429)

Statement	Disagree/strongly disagree n (%)	Unsure n (%)	Agree/strongly agree n (%)
Increasing appropriate use of tPA will:			
Save lives	185 (43.1)	155 (36.1)	89 (20.7)
Not result in unnecessary adverse events	267 (62.3)	102 (23.8)	60 (14.0)
Improve the odds of independent survival for stroke patients	109 (25.4)	150 (35.0)	170 (39.6)
The evidence underpinning tPA use:*			
Is strong when administered within 4.5 h of stroke onset	226 (54.4)	119 (28.7)	70 (16.8)
Indicates that the benefits outweigh the risks if the treatment protocol is followed	134 (32.3)	107 (25.8)	173 (42.0)
Is strong enough to warrant the use of this treatment	141 (34.0)	119 (28.7)	155 (37.4)

Number of observations varies across items due to missing data.

* n = 415.

23.7% of respondents agreed they “have no concerns surrounding the legal implications of tPA use”, and 8.9% agreed they “have no concerns surrounding patient complications of tPA use”. When asked “What would influence your views on the use of tPA in acute stroke?” 83.8% of participants indicated additional trials of tPA, 60.3% reported research conducted by ED staff, 47.6% said guidance from an expert in the use of tPA for acute stroke, and 43.8% indicated guidance from a professional colleague would change their views.

### Factors associated with agreement with evidence for tPA

No physician factors were significantly associated with a high level of agreement with statements supporting tPA use in stroke (Table 4). The perceived routine use of tPA by the head of ED was significantly associated with a high level of agreement with the evidence supporting tPA use (OR 3.87, 95% CI 1.49–10.04, p = 0.01) (Table 5).

**Table 4:** Individual factors associated with high agreement with the evidence for tPA use in acute stroke care

Factors	Crude odds ratio (95% CI)	Adjusted odds ratio (95% CI)	Adjusted p-value
Age	1.01 (0.98, 1.04)	1.06 (0.99, 1.13)	0.09
Gender			
Male	1.21 (0.69, 2.12)	0.96 (0.48, 1.92)	0.91
Female			
Role			
Other vs Emergency physician trainee	0.50 (0.06, 4.27)	0.60 (0.06, 6.02)	0.91
Emergency physician vs Emergency physician trainee	0.91 (0.53, 1.55)	0.92 (0.32, 2.65)	
Years worked in emergency care			
5 years or less vs 16 years or more	1.18 (0.55, 2.50)	2.37 (0.42, 13.31)	0.38
6–10 years vs 16 years or more	0.94 (0.48, 1.87)	1.92 (0.47, 7.85)	
11–15 years vs 16 years or more	1.27 (0.66, 2.44)	2.30 (0.90, 5.92)	
Does your role at the hospital include responsibility for determining the care that is provided to stroke patients?			
Yes	1.23 (0.62, 2.44)	0.92 (0.41, 2.07)	0.84
No			
Does your role at the hospital include deciding which patients receive tPA?			
Yes	1.60 (0.90, 2.85)	1.64 (0.89, 3.04)	0.11
No

**Table 5:** Workplace factors associated with high agreement with the evidence for tPA use in acute stroke care

Factors	Crude odds ratio (95% CI)	Adjusted odds ratio (95% CI)	Adjusted p-value
How many ischaemic stroke patients are seen by the emergency department every fortnight	1.00 (0.98, 1.02)	0.99 (0.97, 1.02)	0.63
The proportion that are referred to a stroke care unit or neurology department	1.00 (0.99, 1.00)	0.99 (0.98, 1.00)	0.28
The proportion of ischaemic stroke patients who are currently treated with thrombolysis at the hospital	1.00 (0.99, 1.02)	1.01 (0.99, 1.03)	0.32
Does the hospital provide tPA treatment to eligible ischaemic stroke patients?			
Yes	1.08 (0.58, 2.00)		.
No			
Does the hospital have arrangements in place to receive pre-hospital notification of stroke patients from the ambulance service?			
Yes	1.19 (0.69, 2.06)	1.02 (0.48, 2.18)	0.95
No			
Does the hospital have a dedicated Stroke Care Unit?			
Yes	1.02 (0.57, 1.84)	0.72 (0.25, 2.03)	0.53
No			
Does the hospital have an Intensive Care Unit?			
Yes	0.78 (0.28, 2.23)	2.46 (0.24, 24.87)	0.45
No			
Does the hospital have advanced imaging facilities (perfusion CT and MRI)?			
Yes	1.16 (0.57, 2.36)	2.28 (0.62, 8.38)	0.21
No			
The proportion of the emergency physicians at your hospital who routinely administer tPA treatment for eligible ischaemic stroke patients			
None	0.63 (0.35, 1.13)	0.96 (0.43, 2.11)	0.91
Less than half/About half/Most/All			
Does the head of emergency department routinely administer tPA treatment for eligible ischaemic stroke patients?			
Yes	4.05 (1.88, 8.72)	3.87 (1.49, 10.04)	0.01
No/I don’t know			
State			
NSW vs TAS	6.08 (0.77, 48.03)		
Vic vs TAS	4.09 (0.52, 32.46)		
QLD vs TAS	6.92 (0.87, 55.34)		
SA vs TAS	4.50 (0.45, 44.55)		
WA vs TAS	2.25 (0.22, 23.46)		
NT vs TAS	0.00 (0.00, I)		
ACT vs TAS	10.80 (0.91, 127.75)		

4th and 11th factors could not be included in the adjusted logistic regression model due to zero counts in some categories.

## Discussion

Even when allowing for the low response rate, study findings suggest a sizeable minority of Australian emergency physician fellows and trainees do not agree with statements supporting use of tPA in the treatment of acute stroke. Consideration of the participating physician and workplace characteristics may suggest that physicians from tertiary hospitals may be over-represented here, therefore, it is likely the majority of results apply to this sub-group.

### Physician agreement with evidence for tPA

As emergency physicians are often the first contact for the in-hospital care of stroke patients, their attitudes towards tPA are a critical factor in the use and non-use of this treatment. Emergency physicians help shape treatment protocols and as such, their perceptions influence the attitudes of others [16]. Given the utility of tPA in significantly reducing disability associated with stroke [5], it is interesting that less than half (39.6%) of respondents agreed that appropriate use of tPA will improve the odds of independent survival for stroke patients.

Only a small proportion of participants agreed the evidence underpinning tPA use is strong when administered within 4.5 h of stroke onset (16.8%), and that the evidence is strong enough to warrant use (37.4%). This is despite approval from the Australian Therapeutic Goods Administration for tPA use, and recommendations in the current Australian clinical practice guidelines [5]. Use of tPA is also approved up to 4.5 h post-stroke onset in the UK by the Medicines and Healthcare Products Regulatory Agency, and recommended up to 4.5 h by AHA and ASA [15]. These results may be indicative of the influence of authoritative emergency-specific bodies that openly do not support the use of tPA by emergency physicians.

Previous studies have identified limited acceptance of the evidence for tPA use in acute stroke [17]. Scott’s study of emergency physicians in the US found 49% agreed the science regarding the use of tPA in stroke is convincing [11]. Additionally, Wang reported 72% of responders recognised tPA is the preferred treatment for acute stroke, and 59% were aware of the limited time-window for administration [16]. While Wang’s outcomes may be biased due to the sampling procedure employed, and the inclusion of residents within family practice, internal medicine and neurology [16], these results highlight a lack of awareness regarding clinical practice guidelines for tPA use in acute stroke care.

Almost half of the respondents in our survey did not agree with any statements supporting tPA use in acute stroke, with only 20% of the respondents having “High agreement” with the literature. In order for tPA treatment to become widely accepted and adopted in emergency settings that have the necessary facilities, beliefs and attitudes towards treatment need to be in accordance with best-practice recommendations.

### Reasons behind physician views of tPA

Research appears to be the key feature influencing attitudes of tPA. A high proportion of respondents reported ‘additional clinical trials of tPA’ (83.8%), and ‘research conducted by emergency department staff’ (60.3%) would influence their views. Indeed, there have been calls for additional clinical trials of tPA treatment to be conducted [18], leading to re-analysis of the National Institute of Neurological Disorders and Stroke trial data [19], and the spread of uncertainty towards use of this therapy [20]. However, given the existing evidence-practice gap, serious consideration about whether additional trials of tPA will actually shift attitudes and practice surrounding the use of tPA, is crucial in determining the next steps forward for the implementation of this therapy. While Scott’s trial targeting hospital staff failed to produce a significant increase in use of tPA for stroke, the authors recognise that additional strategies to increase treatment are required [21].

### Factors associated with agreement with evidence for tPA

Respondents were nearly four times more likely to have high agreement with the evidence supporting tPA use in acute stroke if they perceived their head of ED administers tPA treatment to eligible patients. This result is supported by the 43.8% of respondents who indicated guidance from a professional colleague would influence their views on the use of tPA. While no other studies have examined this relationship, one study found the presence of “uncompromising, individual clinical leadership” in a hospital setting was significantly associated with the likelihood of receiving tPA [22]. Results are indicative of the power of social influence and modelling in changing health providers’ attitudes and behaviour [23]. Social influences play an important role in the implementation of new behaviours, and by targeting provider knowledge, attitudes and social norms, opinion leaders can aid adoption of new practices [24]. Demonstrating or modelling new skills, can build both skill and confidence to perform a desired behaviour [25]. Local opinion leaders in hospital settings can also be effective in promoting evidence-based practice [23].

## Limitations

The study yielded a low response rate (13%), limiting the generalizability of the sample and power of the study. It is possible that only individuals with a strong opinion about tPA use responded. However, low response rates among health-care providers is common and our results are not dissimilar to other online surveys of physicians [26–29]. Hospital characteristics were obtained via self-report and therefore may not be accurate. Hospital location was not obtained, therefore it is not clear how representative results are of physicians working in urban vs rural hospitals.

The survey did not measure respondents’ knowledge of guidelines or criteria for tPA use in stroke, or whether individual physicians had previously administered or were likely to administer tPA. These items may have been associated with high agreement with evidence supporting tPA use as previous use of tPA for stroke is independently associated with a willingness to use tPA [10]. Future studies should measure these constructs. While it is acknowledged that there is conflicting evidence on the use of tPA for acute stroke, the study was specifically designed to assess perceptions on the use of, and evidence supporting, tPA. The terms ‘appropriate’ and ‘treatment protocol’ were used in a number of survey items, assuming a shared meaning of being in accordance with clinical practice guidelines [5]. In addition, as administration of tPA within 6 h of stroke onset significantly increases the odds of being alive and independent at follow-up [4], the term ‘save lives’ was intended to refer to an improvement in quality of life and DALYs, rather than a reduction in mortality. However, as these terms, along with the word ‘unnecessary’, were not defined within the survey, interpretation may differ among respondents.

Finally, 345 NZ fellows and trainees within ACEM were accidently sent the invitation email by ACEM. Although the total number of NZ members has not been included in the denominator, results may contain a number of NZ responders (estimated maximum of 1.4% of responders). The invitation to NZ members was rescinded immediately and is unlikely to have influenced results.

## Conclusions

This was the first study to examine rates of agreement with evidence supporting tPA use in acute stroke care, as well as the individual and hospital factors associated with agreement with the evidence among Australian emergency physicians. Our results correspond with previous international literature, finding low rates of agreement with particular published literature on the potential benefits of tPA use among responding emergency physicians.

Study results demonstrate that attitudes among emergency health-care providers may in fact be one of the factors limiting tPA administration rates. Agreement with clinical practice guidelines for stroke is likely to be necessary if tPA treatment is to become widely adopted in hospitals possessing the appropriate facilities. Future research should explore strategies to increase participation in research among specialist physicians to overcome low response rates and increase the generalisability of study findings. In addition, examination of the effectiveness of health-provider targeted interventions with a focus on social influence and modelling to increase physician agreement with clinical practice guidelines is an area in need of further research. Our findings that the opinions and actions of ED heads and professional colleagues influence emergency physicians’ views may be valuable information to clinical leaders and shape their practice in ensuring health-care provider adherence to clinical guidelines.

Emergency physicians’ perceptions represent a potentially modifiable barrier to the use of tPA treatment. An increase in emergency physician agreement with the literature regarding tPA use in acute stroke may have the potential to produce substantial benefits for stroke patients.

### Availability of supporting data

The data set supporting the results of this article is included within the article and its additional file.

## Additional file

Additional file 1: Perceptions of the use of tissue Plasminogen Activator (tPA) in acute stroke care among emergency physicians.

## Abbreviations

AHA: American Heart Association
ASA: American Stroke Association
ACEM: Australasian College for Emergency Medicine
ACT: Australian Capital Territory
DALYs: Disability-adjusted life years
ED: Emergency Department
F.A.S.T: Face, Arm, Speech, Time
ICU: Intensive Care Unit
NSF: National Stroke Foundation
NSW: New South Wales
NZ: New Zealand
NT: Northern Territory
QLD: Queensland
SA: South Australia
SCU: Stroke Care Unit
TAS: Tasmania
tPA: tissue Plasminogen Activator
UK: United Kingdom
US: United States
VIC: Victoria
WA: Western Australia
