# Supplementary material for: Agreement with evidence for tissue Plasminogen Activator use among emergency physicians: a cross-sectional survey
Source: BMC Res Notes. 2015 Jun 26;8:267. doi: 10.1186/s13104-015-1242-5 (PMC4482289; doi:10.1186/s13104-015-1242-5)
Supplement: Additional file 1: — Perceptions of the use of tissue Plasminogen Activator (tPA) in acute stroke care among emergency physicians. [file 13104_2015_1242_MOESM1_ESM.pdf]

THE UNIVERSITY OF NEWCASTLE AUSTRALIA

Perceptions of the use of tissue Plasminogen Activator (tPA) in acute stroke care among emergency physicians.

We are interested in your views on the use of tissue plasminogen activator (tPA) and about the processes for acute stroke care which are implemented in your work setting. Results of this survey will help develop service improvements for stroke care, by informing an NHMRC funded trial which aims to improve care for acute stroke patients.

The research team
This project is conducted by Laureate Professor Rob Sanson-Fisher, Dr Mariko Carey, Associate Professor Christine Paul, Dr Carolyn Hullick, and Miss Alice Grady from the University of Newcastle. The research is part of Alice Grady's PhD studies at the University of Newcastle. Alice is supervised by Rob Sanson-Fisher, and Mariko Carey from the School of Medicine and Public Health.

Who is invited to participate in this survey?
All fellows and trainees of the Australasian College for Emergency Medicine (ACEM) in Australia are invited to participate.

What will you be asked to do?
You will be asked to complete the following online survey. The survey is to obtain your opinions on the use of tPA and the processes in place for stroke care at the hospital where you work. The survey should take about 10 minutes to complete. You can begin the survey by pressing "Next".

What are the risks and benefits of participating?
We cannot promise you any benefits from participating. However, your answers on the survey will inform future interventions aiming to improve care for acute stroke patients. It is not expected that you will be exposed to any risks by taking part in this study.

What choice do you have?
Participating in this survey is entirely voluntary. Your decision to participate (or not) will not disadvantage you and will not affect your professional relationship with the ACEM or the research team. If you do complete the survey your answers cannot be withdrawn as your data will not be identifiable.

How will your privacy be protected?
All information you provide will be anonymous. That is, it will not be possible to identify you or your workplace from your answers. If you choose to complete the survey, your information will be kept confidential. Data will be retained for at least 7 years after completion of the research project. The data will be kept in locked cabinets and electronic files which will be securely protected so that only the research team will have access to these data.

The online survey is hosted by Survey Monkey. As responses are anonymous, only the information you provide in the survey will be stored by Survey Monkey. Secure Sockets Layer (SSL) technology will be used to protect your information, using both server authentication and data encryption, to ensure that your data is safe, secure, and available only to authorized persons. Further information regarding the security of data held by Survey Monkey can be found at www.surveymonkey.com/privacypolicy.aspx

How will the information collected be used?
Your answers on the survey will inform future interventions aiming to increase best practice stroke care for acute stroke patients.

The study results will be widely publicised via the general media and consumer organizations and made available on a publicly accessible website. A copy of the project report will be provided to the ACEM. Conference presentations and peer-reviewed journal publications will also be used to disseminate study results. Data will only be reported in aggregated form. Results collected in this study will form part of Alice Grady's PhD studies.

***What do you need to do to participate*?**
Please read this information and be sure you understand its contents. Completion of the survey will be considered implied consent. If there is anything you do not understand, or you have questions, contact the researcher.

If you would like to participate, please press "Next". A reminder email will be sent to all potential participants in 2 weeks time.

Further information
If you have any questions or would like further information, please contact Prof Rob Sanson-Fisher on 02 49 138169 or Alice Grady on 02 49 138312. Thank you for taking the time to consider this invitation.

Concerns about this research

This project has been approved by the University's Human Research Ethics Committee, Approval No. H-2011-0288.

Should you have concerns about your rights as a participant in this research, or you have a complaint about the manner in which the research is conducted, it may be given to the researcher, or, if an independent person is preferred, to the Human Research Ethics Officer, Research Office, The Chancellery, The University of Newcastle, University Drive, Callaghan NSW 2308, Australia, telephone (02) 49216333, email Human-Ethics@newcastle.edu.au.

Please rate the extent to which you agree / disagree with the following statements by selecting the appropriate response. If you work at more than one hospital, please think about your main place of employment when answering these questions. Once you have rated all the statements, please click "Next".

	Strongly disagree	Disagree	Unsure	Agree	Strongly agree
**I can:**					
Accurately identify stroke patients	1	2	3	4	5
Confidently interpret brain imaging scans	1	2	3	4	5
Accurately identify which stroke patients may be eligible for tPA	1	2	3	4	5
**This hospital has a policy for:**					
The management of stroke patients	1	2	3	4	5
Rapid referral of suspected stroke patients from ED to stroke specialists	1	2	3	4	5
Rapid access to imaging for suspected stroke patients	1	2	3	4	5
Administration of tPA when appropriate	1	2	3	4	5
**This hospital has:**					
Agreed indicators for quality of stroke care	1	2	3	4	5
Goals for improving performance on stroke care indicators	1	2	3	4	5
**Increasing the appropriate use of tPA will:**					
Save lives	1	2	3	4	5
Not result in unnecessary adverse events	1	2	3	4	5
Improve the odds of independent survival for stroke patients	1	2	3	4	5

	Strongly disagree	Disagree	Unsure	Agree	Strongly agree
**The evidence underpinning tPA use:**					
Is strong when administered within 3 hours of stroke onset	1	2	3	4	5
Is strong when administered within 4.5 hours of stroke onset	1	2	3	4	5
Indicates that the benefits outweigh the risks if the treatment protocol is followed	1	2	3	4	5
Is strong enough to warrant the use of this treatment	1	2	3	4	5
Indicates a theoretical benefit that is difficult to achieve in practice	1	2	3	4	5
**Respected and influential members of this hospital:**					
Endorse the use of tPA	1	2	3	4	5
Actively model best practice stroke care to all staff	1	2	3	4	5
**I have:**					
Seen tPA administered to stroke patients on several occasions	1	2	3	4	5
Received interactive training in tPA administration	1	2	3	4	5
Undergone competency-based assessment for stroke protocol(s)	1	2	3	4	5
Undergone competency-based assessment for tPA use	1	2	3	4	5
Individual performance goals related to stroke care	1	2	3	4	5
No concerns surrounding the legal implications of tPA use	1	2	3	4	5
No concerns surrounding patient complications of tPA use	1	2	3	4	5

	Strongly disagree	Disagree	Unsure	Agree	Strongly agree
**I regularly:**					
Have the opportunity to develop my skills in stroke care	1	2	3	4	5
Treat acute stroke patients	1	2	3	4	5
Have the opportunity to treat stroke cases of varying complexity	1	2	3	4	5
**Respected and influential members of this hospital monitor:**					
My performance of stroke care	1	2	3	4	5
The hospitals' performance on key stroke care indicators	1	2	3	4	5
Actions which are inconsistent with guideline care for stroke patients	1	2	3	4	5
The proportion of eligible stroke patients who receive tPA	1	2	3	4	5
**This hospital has a system in place for:**					
Implementing action plans for improving performance for stroke care	1	2	3	4	5
Reviewing outcomes of quality improvement plans for stroke care	1	2	3	4	5
Entering data from all patients treated with tPA into a central register	1	2	3	4	5
**I am regularly given:**					
Individual performance feedback following relevant cases of acute stroke	1	2	3	4	5
**This hospital regularly receives feedback on:**					
Its performance on stroke care indicators	1	2	3	4	5
Its performance on stroke care indicators compared to other hospitals	1	2	3	4	5

	Strongly disagree	Disagree	Unsure	Agree	Strongly agree
**Excellence in stroke care performance is rewarded at:**					
The hospital level	1	2	3	4	5
An individual level	1	2	3	4	5
**If I do not follow tPA protocol, there are negative consequences for:**					
Me	1	2	3	4	5
The hospital	1	2	3	4	5
The patient	1	2	3	4	5
**To help me follow stroke care protocol there are:**					
Checklists/ decision aids to help me identify and triage a possible stroke case	1	2	3	4	5
Checklists/ decision aids to help me identify stroke patients eligible for tPA	1	2	3	4	5
Checklists/ decision aids to help me interpret imaging for a possible stroke case	1	2	3	4	5
**At all times I have immediate access to:**					
Advice from a senior colleague in managing stroke	1	2	3	4	5
Brain imaging facilities and staff trained to interpret images	1	2	3	4	5
A multidisciplinary acute care team trained in the delivery and monitoring of tPA	1	2	3	4	5

Section B: About You and Your Workplace (please select)

This next section is about you and your workplace. Once you have answered all questions, please click "Next".

1. I am

- ☐ Male
- ☐ Female

2. How old are you?

________years

3. How many years have you worked in emergency care?

- ☐ 5 years or less
- ☐ 6-10 years
- ☐ 11-15 years
- ☐ 16 years or more

4. Would any of the following influence your views on the use of tPA in acute stroke? Please select ‘none’ or as many as apply.

- ☐ Guidance from a professional colleague
- ☐ Guidance from an expert in tPA for acute stroke care
- ☐ Additional clinical trials of tPA
- ☐ Research conducted by Emergency Department staff
- ☐ Other, please specify ___________________

The following questions are about the hospital that you work at. If you work at more than one hospital, please think about your main place of employment when answering these questions. Once you have answered all questions, please click "Next".

5. In what state is the hospital?

- ☐ New South Wales
- ☐ Victoria
- ☐ Queensland
- ☐ South Australia
- ☐ Western Australia
- ☐ Northern territory
- ☐ Australian Capital Territory
- ☐ Tasmania

6. What is your role within the hospital?

- ☐ Emergency physician
- ☐ Emergency physician trainee
- ☐ Other, please specify _______________

7. Does your role at the hospital include responsibility for determining the care that is provided to stroke patients?

- ☐ Yes
- ☐ No

8. Does the hospital have arrangements in place to receive pre-hospital notification of stroke patients from the ambulance service?

- ☐ Yes
- ☐ No

9. Please estimate on average how many ischaemic stroke patients are seen by the emergency department every fortnight. Please give your best estimate.

_________patients

10. Of the stroke patients seen by the emergency department, please estimate the proportion (%) that are referred to a stroke care unit or neurology department. Please give your best estimate.

________%

11. Does the hospital have a dedicated Stroke Care Unit?

- ☐ Yes
- ☐ No

12. Does the hospital have an Intensive Care Unit?

- ☐ Yes
- ☐ No

13. Does the hospital have advanced imaging facilities (perfusion CT and MRI)?

- ☐ Yes
- ☐ No

14. Does the hospital provide tPA treatment to eligible ischaemic stroke patients?

- ☐ Yes
- ☐ No → End of survey

If yes to 14

15. Please estimate approximately what proportion (%) of ischaemic stroke patients are currently treated with thrombolysis at the hospital. Please give your best estimate.

________%

16. Does your role at the hospital include deciding which patients receive tPA?

- ☐ Yes
- ☐ No → Go to 18

If yes to 16

17. Please estimate approximately what proportion (%) of eligible ischaemic stroke patients you treat with thrombolysis. Please give your best estimate.

________%

18. What proportion of the emergency physicians at your hospital routinely administer tPA treatment for eligible ischaemic stroke patients?

- ☐ None, tPA treatment is administered by other specialists at the hospital
- ☐ Less than half
- ☐ About half
- ☐ Most
- ☐ All

19. Does the head of emergency department routinely administer tPA treatment for eligible ischaemic stroke patients?

- ☐ Yes
- ☐ No
- ☐ I don't know

Thank you very much for taking the time to complete the survey. Your answers will help in identifying the views of emergency physicians regarding use of tPA use in acute stroke care.
